# The twisted survivin connection to angiogenesis

**DOI:** 10.1186/s12943-015-0467-1

**Published:** 2015-11-19

**Authors:** C. Sanhueza, S. Wehinger, J. Castillo Bennett, M. Valenzuela, G. I. Owen, A. F. G. Quest

**Affiliations:** Cellular and Molecular Physiology Laboratory (CMPL), Division of Obstetrics and Gynecology, School of Medicine, Faculty of Medicine, Pontificia Universidad Católica de Chile, Santiago, 8330024 Chile; Interdisciplinary Excellence Research Program on Healthy Aging (PIEI-ES), Universidad de Talca, Talca, Chile; Cellular Communication Laboratory, Center for Molecular Studies of the Cell (CEMC), Program of Cell and Molecular Biology, Institute of Biomedical Sciences (ICBM), Faculty of Medicine, Av. Independencia 1027, Santiago, Chile; Advanced Center for Chronic Diseases (ACCDiS), Santiago, Chile; Facultad de Ciencias Biológicas & Center UC Investigation in Oncology, Pontificia Universidad Católica de Chile, Santiago, Chile

**Keywords:** Survivin, Hypoxia, Cell survival, ROS, VEGF, Angiogenesis, Oxidative stress, Cancer

## Abstract

Survivin, a member of the inhibitor of apoptosis family of proteins (IAPs) that controls cell division, apoptosis, metastasis and angiogenesis, is overexpressed in essentially all human cancers. As a consequence, the gene/protein is considered an attractive target for cancer treatment. Here, we discuss recent findings related to the regulation of survivin expression and its role in angiogenesis, particularly in the context of hypoxia. We propose a novel role for survivin in cancer, whereby expression of the protein in tumor cells promotes VEGF synthesis, secretion and angiogenesis. Mechanistically, we propose the existence of a positive feed-back loop involving PI3-kinase/Akt activation and enhanced β-Catenin-TCF/LEF-dependent VEGF expression followed by secretion. Finally, we elaborate on the possibility that this mechanism operating in cancer cells may contribute to enhanced tumor vascularization by vasculogenic mimicry together with conventional angiogenesis.

## Background

Survivin (BIRC5) is a member of the IAP family that participates in cell division, apoptosis inhibition and angiogenesis [1–3]. In humans, survivin is widely expressed in development but generally not present in adult tissues [4]. However, upon malignant transformation the protein survivin is commonly re-expressed [5] leading to the promotion of cell survival, proliferation and metastasis. For these reasons, survivin is considered a potentially interesting target for cancer therapy [2]. In tumors, the expression of survivin and VEGF are closely linked during tumor growth and angiogenesis, and are detected in several types of cancer [6–9]. In this review, we will focus predominantly on the role of survivin in angiogenesis. Recent findings from our group identified a novel pathway by which survivin promotes VEGF (vascular endothelial growth factor) expression in cancer cells, hence promoting angiogenesis, a rate-limiting step in tumor growth. Also, we will elaborate on the relevance of this pathway in the context of hypoxia, reactive oxygen species (ROS) formation and β-Catenin signaling. Finally, we will consider the possibility that survivin may also be relevant to the process of vasculogenic mimicry suggested to occur in regions of tumors that lack endothelial cell-mediated vascularization.

### Survivin and cell survival in hypoxia: hypoxia-induced factors

#### Hypoxia

Under normal physiological conditions, cells are supplied with oxygen at concentrations ranging from 1-13 % O_2_ depending on the tissue [10, 11]. However, under pathological conditions, as is the case in cancer, tissue oxygenation is severely impaired due to insufficient vascularization resulting in a condition known as hypoxia [10, 12]. In patients, hypoxia has been reported in most solid tumors, including prostate [13], pancreas [14], head and neck [15], breast [16], kidney [17] and liver cancer [18]. Beyond the immediate responses linked to lack of oxygen and nutrients, including the induction of Hypoxia-Induced Factors (HIFs), autophagy and the unfolded protein response (UPR), tumor hypoxia is also associated with resistance to chemotherapy, metastasis and reduced patient survival [19–23]. Unfortunately, these adaptive mechanisms enable cancer cells to adjust to low oxygen concentrations, proliferate and ultimately disseminate to distant sites. In this review, we will center the discussion mainly on HIF-related adaptive responses.

In normal tissues, blood vessels are characterized by a well-defined hierarchical organization and reside in close proximity of target cells in order to ensure a constant oxygen and nutrient supply. However, in tumors an imbalance between oxygen consumption/delivery due to excessive proliferation results in hypoxia [24], a highly dynamic process which involves both periods of chronic and cyclic hypoxia [25]. Several factors contribute to and exacerbate the hypoxic microenvironment. Typically, the tumor blood vessels are disorganized, aberrantly branched and frequently distant from the tumor cells. Also, the blood vessels are more leaky leading to blood deviation and increased compensatory blood flow bypassing tumor blood vessels, thereby reducing oxygen levels even further. In addition, tumor blood vessels are characterized by fragile capillary walls and retarded blood flow, which again accentuates hypoxia [24, 26]. Lower vascular density, inherent limitations in oxygen diffusion, erythrocyte hardening and increased blood viscosity all contribute to reducing the blood flow, and thus generating an extravascular hypoxic niche [27].

#### Cellular adaptation to hypoxia by HIFs

As previously indicated, cellular responses to hypoxia are complex, and dependent on the severity of hypoxia and the duration of the stimulus. Under conditions of hypoxia (O_2_ 0.1 %-5 %), cells adapt by the activation of the HIF transcription factors that are responsible for metabolic adaptation, pH control and the neovascularization process [28]. HIFs are heterodimeric transcription factors of the bHLH family, comprised of an oxygen-dependent (α) and a constitutive subunit β [29]. To date, three oxygen-sensitive isoforms have been described (1α, 2α and 3α) [29–31], of which HIF1α is the best-described isoform whose expression is more ubiquitous in comparison to HIF2α or HIF3α [32–34].

In normoxia, prolyl-hydroxylase enzymes (PHDs) hydroxylate HIFα on key proline residues that are recognized by the Von Hippel Lindau (VHL) factor, an E3 ubiquitin ligase that promotes proteasomal HIFα degradation [35–37]. Reduced oxygen levels in hypoxia prevent HIFα hydroxylation by PHDs thereby stabilizing HIFα, which then translocates to the nucleus to form an heterodimer with HIF1β and bind to the *hypoxia-responsiveness elements* (*HRE*, core sequence 5´-(A/G) CGTG-3´) present in the promoter sequence of a large number of target genes [38–41]. Although both HIF1α and HIF2α bind to the same HRE promoter sequences, the increase in expression of specific target genes depends on HIF interaction with other factors. Thus, HIF target genes are classified according to their HIF subunit dependence as HIF1α-, HIF2α- or HIF1α/HIF2α-dependent [42]. Recently, the interactions between HIF1α/STAT3 and HIF2α/USF2 have been reported to activate the expression of a unique subset of target genes [43–46]. In addition, the activation of HIF-associated Factor (HAF), a HIF1α-specific target gene, permits the transition from HIF1α- to HIF2α-dependent adaptation processes during prolonged hypoxia [12, 47]. In general terms, the HIF1α response appears to be important to trigger adaptive alterations in cellular metabolism [46, 48], while HIF2α-dependent responses are essential to induce cell proliferation in hypoxia, in a manner dependent on c-myc [49, 50]. In summary, exposure to hypoxia triggers a highly complex array of responses that is mediated by different HIFs and the interaction with additional factors.

#### HIF1α stabilization due to ROS production

While proline hydroxylases (PHD) have been widely regarded as the primary oxygen sensors mediating cellular responses to hypoxia, available evidence indicates that mitochondria also respond to low oxygen tension, generating ROS, which then activate intracellular pathways to control the expression of several pro-survival genes [51]. Indeed, chronic treatment of Hep3B cells with ethidium bromide was shown to generate respiration-deficient p^0^ cells lacking functional mitochondria and, using this model, the authors then showed that mitochondria-dependent signaling processes involving ROS are required in hypoxia to promote HIF1α stabilization. In addition, mitochondrial ROS were found to be necessary for both HIF1α-DNA binding and the induction of HIF1α-mediated expression of Erythropoietin (EPO), VEGF, as well as glycolytic enzymes [52]. Furthermore, mitochondria-derived ROS are not only required, but are also sufficient to initiate HIF1α stabilization during hypoxia, and this effect requires an active mitochondrial complex III [38, 53, 54]. Importantly, ROS released into the cytosol from mitochondria stabilize not only HIF1α, but also HIF2α [53]. Thus, mitochondrial ROS plays an important role in HIFα stabilization and target gene expression in hypoxia.

In addition, HIF1α may also be stabilized in normoxia due to increased ROS production [38]. Importantly, ROS formation, ROS-dependent HIF1α stabilization and increased VEGF levels have been observed under normoxic conditions in hepatoma, lung carcinoma and osteosarcoma cell lines. Moreover, reduced activity of mitochondrial complex II (succinate-ubiquinone oxidoreductase, Sdh) due to diminished activity of the B subunit (ShdB) in such cells, favors tumor cell growth in a HIF1α-dependent manner [55]. Although it is broadly accepted that ROS can modulate HIF1α activity, often the source(s) of ROS and/or the mechanism(s) leading to ROS generation remain controversial. However, it has become increasingly clear that ROS modulate the rate of HIF1α hydroxylation [56]. A possible mechanism to explain these observations includes direct inhibition of PHD or effects of ROS on the levels of ascorbate, Fe(II) or Krebs cycle (TCA) intermediates [57, 58]. Indeed, ROS are not the only modulators of PHD activity. Multiple mitochondrial products, including TCA cycle intermediates, have been shown to favor HIF1α stabilization and cellular responses similar to those observed upon O_2_ depletion [37, 59] For instance, an increase in succinate levels in the absence of ROS in HEK cells leads to HIF1α stabilization and increased VEGF mRNA levels by PHD inhibition in normoxia [60]. Also, Pollard *et al* provided in vivo evidence to support the notion that increased succinate and/or fumarate stabilize HIF1α, possibly through the inhibition of PHD [61].

As tumors develop, tumor cells become increasingly oxygen deprived and need to reprogram their metabolism to adapt. This is achieved initially by decreasing the aerobic respiration rate and increasing glycolytic activity. In this case, increased ROS levels, generated by mitochondrial complex III, stabilize HIF1α via oxidation/inactivation of PHD [62]. Beyond mediating the initial steps in tumor cell adaptation to the hypoxic environment of a growing tumor, ROS formation has also been linked to tumor cell aggressiveness. For instance, Ishikawa *et al* evaluated the contribution of mutations in mitochondrial DNA to metastasis. Using the cybrid technology, they replaced mitochondrial DNA from a cell line with low–metastatic potential by donor mitochondrial DNA from a highly metastatic mouse cell line. Mitochondrial DNA from metastatic cell lines contains mutations, which result in a Complex I-deficient cell with increased ROS production. This exchange of mitochondrial DNA was sufficient to induce a metastatic phenotype in recipient cells, via ROS formation with elevated HIF1α and VEGF production [63]. Taken together, these observations point towards the existence of an intricate connection between mitochondrial function/ROS levels and HIF1α activation in cancer.

Moreover, it is well established that hypoxia is associated with resistance to chemotherapy. Recently, hypoxia-driven ROS were shown to trigger a biphasic, redox-dependent response that protects cells against etoposide-induced apoptosis. In this case, both mitochondrial- (mtROS) and NADPH oxidase-derived ROS (noxROS) cooperate in HIF1α stabilization, VEGF expression and cell survival. Furthermore, the authors proposed the existence of a VEGF-dependent autocrine loop that results in redox-mediated, prolonged stabilization of HIF1α [64].

Mitochondrial ROS seem to play a dual role in hypoxia signaling related to malignancy in tumors. Hypoxia (5 % O_2_) generates mROS, which activate both NF-κB through c-Src-mediated phosphorylation of IκB-α on tyrosine residues, and stabilizes HIF1α - and increases VEGF expression. These events promote carcinogenesis by the induction of survival pathways that protect cells in the face of DNA damage and permit tumor progression [58, 65]. Moreover, recently the combined treatment using S13, a Src-specific tyrosine kinase inhibitor, together with paclitaxel, dramatically reduced prostate cancer tumor growth. This effect was attributed to a reduction in ROS production, HIF1α stabilization and *de novo* formation of blood vessels [66]. These findings indicate that ROS favor tumor progression by activating HIF1α and increasing VEGF expression.

### ROS and angiogenesis

Angiogenesis is characterized by the sprouting of new blood vessels from the pre-existing vasculature and is triggered by pro-angiogenic factors, such as fibroblast growth factor (FGF), platelet derived growth factor (PDGF), epidermal growth factor (EGF), hepatocyte growth factor (HGF), Angiopoietins (Ang1, Ang2), TIE1 and TIE2, Ephrins, Neuropeptide Y and the previously mentioned VEGF. This latter family represents the best-characterized group of endothelial growth factors to date [67].

A close relationship exists between angiogenesis and oxidative stress in both physiological and pathological settings [68]. ROS are key mediators of this process that may be produced as a side product of the mitochondrial electron transport reaction, the activation of NADPH oxidases or upon exposure to cytotoxic drugs. ROS are commonly employed in many physiological processes in the cell and thus cannot be considered toxic *a priori*; however, when produced in excess, the oxidative stress generated in cells can contribute to pathological development. Generation of intracellular ROS is associated with VEGF-dependent signaling in endothelial cells [69]. Importantly, tumor growth is strongly dependent on angiogenesis and in the tumor microenvironment, ROS generated by NADPH oxidases increase VEGF secretion in a HIF1α-dependent manner [70]. Also, inflammatory mechanisms are strongly linked to ROS production and angiogenesis. In the wound healing process, neutrophils and macrophages release ROS, which in turn promote VEGF release [71]. Interestingly, in vitro stimulation of angiogenesis has been observed in bovine thoracic aorta exposed to hydrogen peroxide to promote mild oxidative stress [72]. Moreover, it has been suggested that tumor cells promote angiogenesis by releasing large amounts of hydrogen peroxide [73]. The hypothesis that oxidative stress is an important inducer of angiogenesis is further supported by evidence showing that anti-oxidants inhibit angiogenesis. For instance, leptin, a circulating hormone secreted principally by adipocytes, promotes angiogenesis by enhancing VEGF production, while N-acetylcysteine (NAC) blocks leptin-induced VEGF transcription in microvascular endothelial cells [74]. Likewise, diphenyliodonium and apocinin (a NADPH oxidase inhibitor), mannitol and catalase and other radical scavengers, have all been shown to inhibit angiogenesis [75–77]. Furthermore, vascularization in melanomas is inhibited by over-expressing extracellular superoxide dismutase (SOD) [78]. On the other hand, mice lacking NADPH oxidase 2 display impaired VEGF-induced angiogenesis and neovascularization following hind limb ischemia [79].

**Fig. 1:**
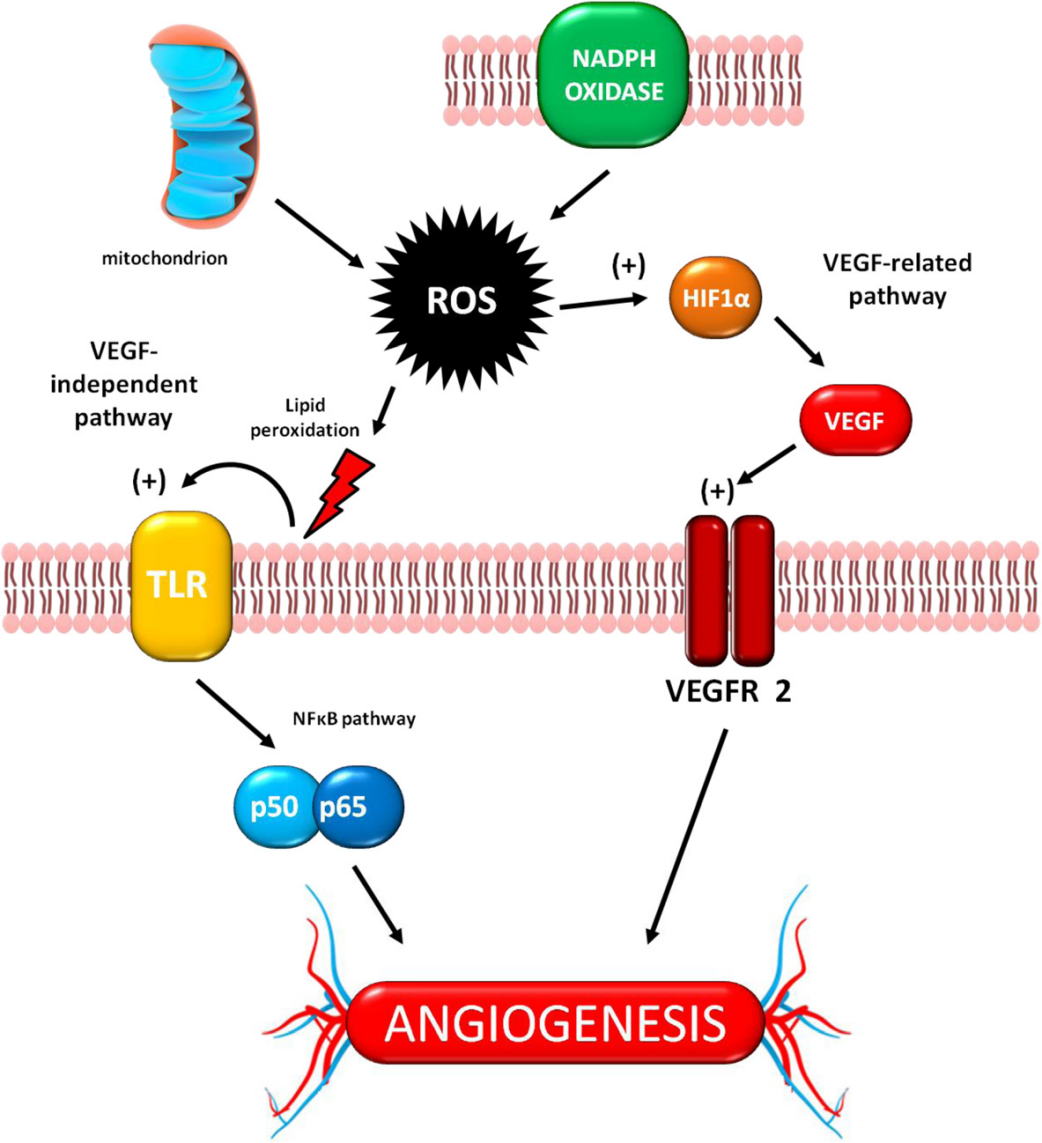
Oxidative stress and angiogenesis. The two main sources of oxidative stress, mitochondria and NADPH oxidases, generate ROS that trigger angiogenesis in two different ways, either by VEGF-related or VEGF-independent pathways – see main text for details. TLR: Toll Like Receptor

The VEGF pathway is modulated by ROS and oxidative stress stimulates VEGF production in several cell types, including endothelial cells, smooth muscle cells and macrophages [68]. ROS enhance angiogenesis by increasing HIF1α, as well as the expression and activity of VEGF receptor-2 (VEGFR2) [69, 70, 80]. This ROS-VEGF connection becomes even more complex when considering that VEGF promotes cell migration and proliferation by increasing intracellular levels of ROS [81]. However, it should be noted that oxidative stress also induces angiogenesis in a VEGF-independent manner by phospholipid oxidization, generating metabolites that act either as ligands or by inducing post-translational modifications (eg. ω-carboxyalkylpirrole: CAP) of proteins within angiogenic signaling pathways. Relevant examples include the Toll like receptor (TLR)2/MyD88 [82] and NFκB activation [83] pathways (Fig. 1).

In summary, the discussion so far of a considerable body of evidence has revealed the existence of an intricate and complex connection between oxidative stress and angiogenesis. The following sections, will focus on highlighting how survivin fits into this already complex picture.

### Survivin

In endothelial cells, the principal targets of HIF-dependent, pro-angiogenic responses are VEGF-A and Survivin [2, 84, 85]. Survivin, also called baculoviral inhibitor of apoptosis repeat-containing 5 (BIRC5), is a member of the inhibitor of apoptosis (IAP) family whose expression greatly favors tumor cell survival through activation of multiple pathways (see Lladser et al [2]). HIF activation in hypoxia triggers different strategies to aid in promoting tumor cell survival. While, VEGF-A is controlled by both HIF1α and HIF2α, survivin expression is solely dependent on HIF1α activation [45, 86–88].

**Fig. 2:**
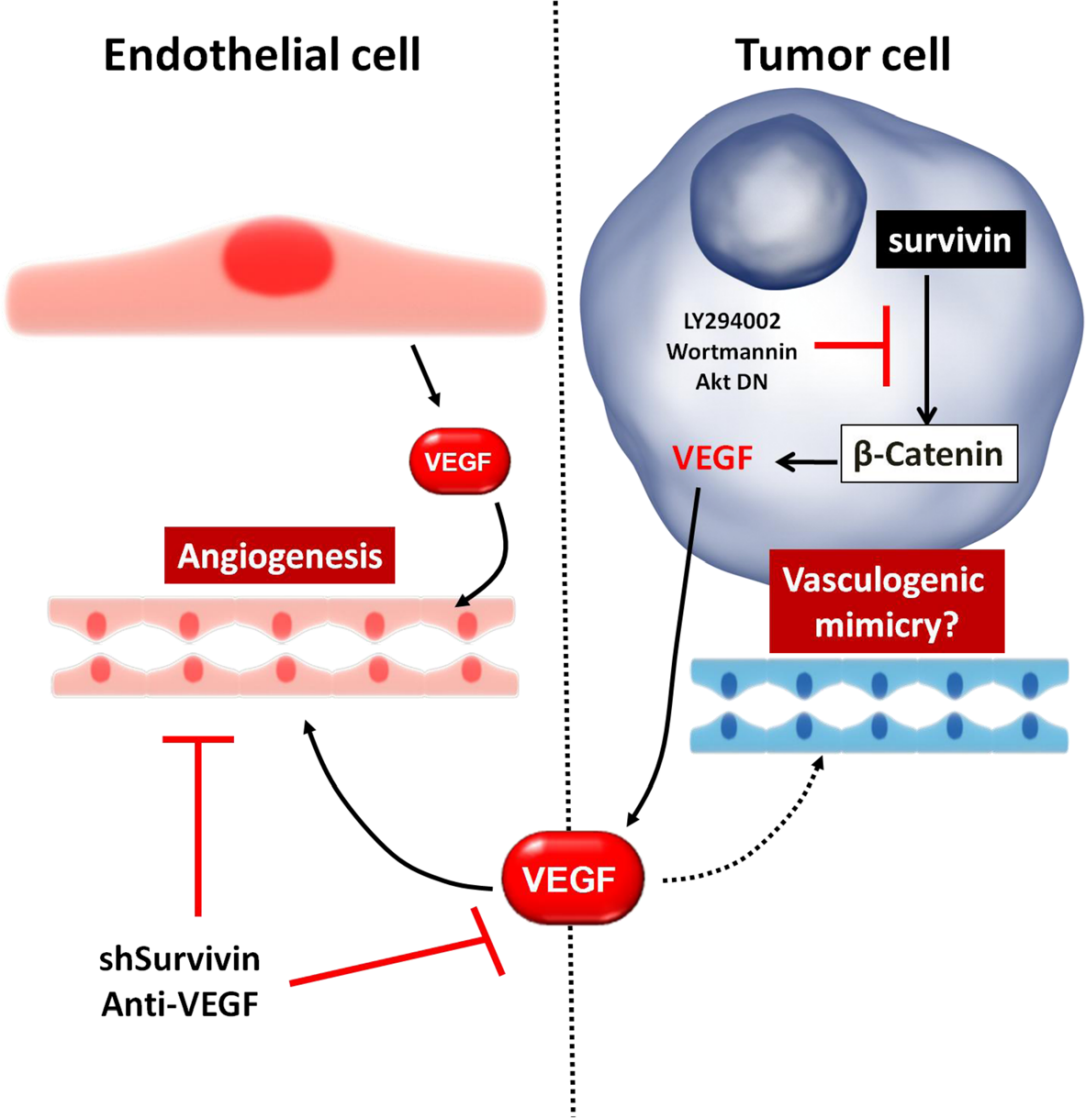
The Survivin/VEGF connection in angiogenesis. Tumor cells overexpressing Survivin induce VEGF synthesis/release in a β-catenin signaling-dependent manner. Liberated VEGF may act on endothelial cells promoting angiogenesis together with endothelial cell secreted VEGF. Alternatively, in tumors with few endothelial cells, survivin-induced VEGF synthesis/release may promote vasculogenic-mimicry. For details, see main text

Survivin is widely expressed in fetal development, but generally then becomes undetectable in normal adult tissues [4], although there are notable exceptions, as is the case for the gastric mucosa [89]. Importantly, however, survivin is commonly re-expressed in human tumors and is required for cancer cell survival [2, 5]. Suppression of apoptosis is a hallmark of the cancer cell that typically becomes genetically unstable, highly proliferative, and resistant to therapy [90]. Survivin, has emerged as a central player in this context due its roles in proliferation, inhibition of apoptosis, metastasis and angiogenesis [1–3, 91]. In hypoxia, HIF1α-targeting reduces survivin expression, thereby compromising cell viability. For instance, inhibition of HIF1α by Echinomycin reduces survivin expression and sensitizes multiple myeloma cells to melphalan-induced apoptosis [92]. In addition, miRNA-mediated HIF1α *knockdown* reduces survivin expression and induces cell death, while survivin overexpression prevents apoptosis in A549 lung cancer cells [93]. Furthermore, shRNA-mediated targeting of HIF1α reduces survivin mRNA and protein expression in the SW480 colon cancer cell line, thereby increasing the apoptotic index and reducing in vivo tumor growth [94]. Finally, in the gastric cancer cell lines SGC7901 and BGC823, survivin is upregulated in an AKT/HIF1α dependent manner, and promotes resistance to cisplatin [95]. Taken together, these findings strongly suggest that survivin expression is a downstream target of HIF1α and importantly, that survivin function is required to maintain cell viability in hypoxia. Thus, HIF1α-dependent transcription of survivin may mediate cell survival under the low oxygen conditions commonly associated with tumor growth. In addition, we envisage that increased survivin expression may contribute to VEGF synthesis in hypoxia in a manner dependent on VEGF-A up-regulation by HIF1α and HIF2α. Specifically, as indicated in the schematic (see Fig. 2), recent studies show that the production of VEGF in tumor cells is connected to survivin expression via PI3K/Akt-dependent activation of β-catenin/Tcf-Lef-mediated VEGF transcription [3], as is described below.

#### Survivin and oxidative stress

Survivin overexpression in human cancers is also associated with drug resistance and, interestingly, in some cases resistance to oxidative stress. As referred to previously, ROS can induce HIF1α stabilization and increase VEGF expression; however, there are no reports linking survivin overexpression to ROS-dependent HIF1α stabilization. Alternatively, evidence for a negative correlation between ROS, VEGF production and survivin expression is available. For instance, reduced tumor cell survival has been observed using treatments with 2-cyano-3,12-dioxooleana-1,9-dien-28-oic acid (a synthetic triterpenoid and PPARγ ligand) and its methyl ester [96, 97] together with pro-oxidant concentrations of ascorbic acid (5 mM) [98]. This combination was shown to induce the formation of ROS that, via miRNA induction, suppress Sp-transcription factors leading to a reduction in survivin and VEGF expression. This may be taken to indicate that Survivin/VEGF induction by ROS is strongly dependent on the amount of ROS generated. An excess in ROS production may lead to cell death by reduction of Survivin/VEGF expression. Alternatively, oxidative stress induced by phototherapy can lead to a rapid upregulation of inducible nitric oxide synthase (iNOS), which, in turn, promotes a notable increase in survivin expression as part of a protective response in breast cancer cells [99]. On the other hand, oxidative stress also triggers anti-tumor effects by the down-regulation of anti-apoptotic proteins, such as survivin. Resistance to oxidative stress greatly favors tumor cell survival given that tumors are known to produce large amounts of ROS [73], which contribute to tumor progression by enhancing genetic instability [100]. For instance human hepatoma cells undergo apoptosis In a ROS-dependent manner, when treated with the NF-κB inhibitor dehydroxymethyl-epoxyquinomicin (DHMEQ), due to down-regulation of BCL2, Mcl-1 and survivin [101]. Furthermore, a number of studies correlate pro-oxidant cytotoxic effects of compounds with survivin down-regulation, or, alternatively protective effects against oxidative stress with increased levels of survivin, supporting the idea that survivin contributes significantly to protection against pro-apoptotic oxidative stress [102–104]. On the other hand, oxidative stress may induce apoptosis through survivin down-regulation. As an example, Zinc oxide nanoparticles have been shown to induce oxidative stress in human alveolar adenocarcinoma, and this is linked to the down-regulation of survivin and anti-apoptotic proteins [105].

*Helicobacter pylori* (Hp), a pathogen associated with the development of gastric cancer is known to generate oxidative stress upon infection. Recently, Hp-induced gastritis and damage to the gastric epithelium was linked to loss of survivin expression in the gastric mucosa. Importantly, both loss of survivin and gastric cell line viability was shown to involve enhanced protein degradation via a ROS/Fe-dependent pathway [89, 106]. Taken together, these observations favor the notion that survivin acts as a resistance factor to oxidative stress-induced apoptosis. Furthermore, loss of survivin renders both normal and tumor cells vulnerable to cell death promoting signals.

Thus, although many details remain to be defined, our current understanding of the connections between ROS, HIF1α, VEGF and Survivin point towards the latter is a key point of convergence and a crucial component in determining tumor growth, progression, metastasis and drug resistance.

### The up-regulation of VEGF and survivin in cancer

VEGF is required for neo-vascularization under physiological conditions and is fundamental during tumor formation, proliferation and metastasis [85, 107, 108]. The co-expression of VEGF and survivin has been reported in many types of cancer, including small-cell lung [6], bladder [7], thyroid [8] and nasopharyngeal [9] carcinomas. Accordingly, drugs that antagonize VEGFR function reduce angiogenesis and tumor growth, as well as sensitize cells to apoptosis [109], and therefore hold great therapeutic promise for cancer treatment [110]. Interestingly, the anti-apoptotic effects of VEGF are directly associated with the activation of pro-survival signaling pathways [111]. For instance, anti-apoptotic genes, such as survivin and bcl-2, are upregulated in endothelial cells via β-catenin/Tcf-Lef activation following VEGF treatment in vitro [112]. Furthermore, MAPK/ERK activation by VEGF protects endothelial cells against ceramide-induced death [113]. While hypoxia and subsequently HIF1α stabilization are main factors in VEGF production, other signaling pathways also induce this pro-angiogenic protein. For instance, ischemic pre-conditioning leads to cardioprotection through VEGF, survivin and bcl-2 by activating the β-catenin/Tcf-Lef signaling pathway [114, 115].

The apparent correlation between VEGF and survivin expression in cancer can be explained by the fact that VEGF induces survivin transcription. Survivin expression is controlled at the transcriptional and post-transcriptional level [116, 117], through the PI3K [118–120], mTOR [121], Ras [122] (79), AMPK [123] and Bcl-2/ERK [124] pathways. Extracellular stimuli that activate these pathways include VEGF, EGF, and cytokines [125]. Accordingly, the control of survivin can be attributed to the regulation of a large variety of transcription factors, including p53 [126], STAT3 [127–129], PTEN [130], NF-κB [131, 132], KLF4 [133], KLF5 [134], EGR-1 [135], E2F-1 [136], SP-1 and SP-3 [137], FOXO1 [138], HIF1α [87] and β-catenin/Tcf-Lef [139].

As eluded to before, survivin protects cancer cells in the face of pro-apoptotic stimuli. Moreover, down-regulation of survivin correlates with lower levels of VEGF [140] and reduced angiogenesis [107, 141] in cancer cells. Furthermore, in vivo studies in the zebrafish have confirmed that loss of survivin expression impairs angiogenesis, leading to developmental complications. Interestingly, in this model the authors showed that this defective phenotype could be rescued by VEGF treatment [142, 143], demonstrating thereby in vivo the relevance of this link between survivin, VEGF and angiogenesis. Consistent with this interpretation, survivin overexpression augments the secretion of pro-angiogenic molecules, such as VEGF and bFGF, and promotes angiogenesis in glioma cells in vitro and in vivo [144] or in skin flaps [145]. The molecular mechanisms implicated in this process are discussed below.

In transcriptional regulation, survivin overexpression increases the phosphorylation state and activation of proteins, including the transcriptional factors Sp1 and c-Myc [146]. Additionally, it has been demonstrated that survivin overexpression activates PI3K/AKT signaling and subsequent β-catenin/Tcf-Lef-dependent transcription, which increases the expression of VEGF, among other transcriptional target genes [3]. Importantly, survivin-targeting by a shRNA or inhibition of PI3K/Akt reduces β-Catenin/TCF-Lef transcriptional activation, indicating that survivin modulates β-Catenin/TCF-Lef activity via a PI3K/Akt-dependent pathway. In vivo*,* down-regulation of *s*urvivin was shown to reduce the microvessel density and VEGF expression in B16F10 tumors. Taken together, the data suggest that survivin overexpression in tumor cells promotes angiogenesis by PI3K/Akt-mediated activation of β-Catenin/TCF-Lef-dependent VEGF transcription.

Survivin has also been reported to regulate protein expression at the post-transcriptional level via its ability to reduce caspase activity. As an example, may it suffice to say that survivin enhances p53 degradation by inhibiting the caspase-dependent cleavage of Mdm2 and thereby modulating a cell-cycle checkpoint [147]. During the process of mitosis, survivin enhances the activity of the Aurora B kinase by stabilizing the chromosomal passenger protein Aurora B [146, 148]. The deregulation of the Aurora complex may lead to unequal distribution of genetic information and thus contribute to the aneuploidy observed in cancer cells. Beyond modulating caspase activity, a study evaluating survivin binding partners revealed that 18 % of the estimated interactions occurred with kinases [116], and particularly the down-regulation or inhibition of Aurora B kinase was directly associated with reduced PI3K/AKT phosphorylation [149, 150]. This observation raises the specter that survivin may activate PI3K/Akt-β catenin signaling by stabilizing Aurora B. Furthermore, other reports directly link PI3K/Akt to β-catenin signaling by GSK3β phosphorylation/inhibition and β-catenin stabilization [151–154]. Additionally, a positive feedback loop between β-catenin/Tcf-Lef target genes was also observed for COX-2, where PGE2 regulated survivin expression in hepatocellular and colon carcinoma cells through the EP receptors via the EGFR/PI3K and Gs-axin/β-catenin signaling pathways, respectively [155–157].

These observations can be taken to suggest that survivin-mediated Aurora B stabilization combined with a subsequent positive amplification loop mechanism involving PI3K activation may favor β-catenin TCF/Lef activation and VEGF synthesis. However, further experiments are required to corroborate this intriguing possibility.

### A potential role for survivin in vasculogenic mimicry?

While angiogenesis has long been accepted as a necessity for tumor growth, in the last decade there have been observations indicating that tumors can continue to grow with limited vasculature. The mechanism behind this survival is speculated to be the process of vasculogenic mimicry [158, 159]. The phenomenon of vasculogenic mimicry describes the formation of tubular structures within the tumor that are of cancer cell origin and thus independent of endothelial cells. As with angiogenesis, an underlying mechanism of induction of vasculogenic mimicry seems to be hypoxia. Unsurprisingly, given the similarities with angiogenesis, genes implemented in vasculogenic mimicry are those previously associated with vascular (VE-cadherin), embryonic (Nodal, Notch4), and hypoxia-related (hypoxia-inducible factor, Twist1) signaling pathways [160]. VEGF and its receptor VEGFR type 2 (also called KDR, Flk-1), have been implicated in vasculogenic mimicry [161, 162]. Expression of the ανβ5 integrin also correlated with vasculogenic mimicry and highly aggressive melanoma [163]. Ovarian tumors exhibiting vasculogenic mimicry demonstrated higher expression of β-catenin and VEGF [164]. In hepatocellular carcinoma cells, VEGF-induced vasculogenic mimicry is also reported to involve Myocyte Enhancer Factor 2C (MEF2C) together with β-catenin via the p38 MAPK and PKC signaling pathways [165]. The search for the exact mechanisms and the unique pathways involved in the process is still very much in its infancy [166]; however, as survivin is overexpressed in almost all human cancers [2] it remains to be determined whether survivin participates in tumor cell mediated vasculogenic mimicry. Indeed, some available evidence suggests that survivin could play a role in this process. First, vasculogenic mimicry is known to be associated with higher β-catenin and VEGF expression [164]. Second, tumor hypoxia accelerates the vasculogenic mimicry process [167] and both Survivin and VEGF expression are upregulated by HIFs in hypoxia [6, 45, 87, 88]. Furthermore, in ovarian cancer, hypoxia has been shown to promote vasculogenic mimicry formation by inducing epithelial-mesenchymal transition (EMT) [167]. The relationship between vasculogenic mimicry and EMT has been reported in numerous cancer types including glioma, liver, head and neck, and stomach cancer [168–172]. Further suggesting a role for survivin, the upregulation of survivin and the induction of EMT has been widely reported in both cellular physiology and cancer, as shown in human retinal pigment epithelial cells and in glioblastoma, among many other cell models [173, 174].

Survivin is also reported to be involved in the interplay between CD31 and VE-Cadherin, both implicated in vasculogenic mimicry. In esophageal carcinoma cells, knock-down of HIF1α inhibited vasculogenic mimicry and HIF1α was shown to upregulate VE-cadherin expression [175].

Evidence is also present connecting CD31, VE-Cadherin, β-catenin and survivin in physiological processes. The endothelial cells of CD31 knock-out mice possess reduced VE-cadherin expression with a corresponding increase in levels of survivin [176]. In accordance, confluence and VE-cadherin and β-catenin are reported to negatively regulate the synthesis of survivin in endothelial cells. Using β-catenin null and positive isogenic endothelial cell lines this down-regulation of survivin has been shown to require β-catenin [177]. Moreover, survivin promotes VEGF synthesis/secretion by tumor cells, thereby favoring angiogenesis [3]. Bearing in mind these observations, one may speculate that in poorly vascularized tumor regions, survivin-mediated VEGF synthesis and/or HIF1α mediated survivin and VEGF expression could promote vasculogenic mimicry and thus favor tumor survival (see Fig. 2).

### Survivin expression in human cancers

As eluded to, Survivin plays multiple pleiotropic roles that are important for cancer development and progression. Survivin participates in the cell division process [178–180], protects against cell death via prevention of SMAC/DIABLO release [2] and promotes angiogenesis [3]. Also, survivin participates in the maintenance stemness and promotes cell motility, as well as metastasis [181]. In conjunction, these survivin functions strongly contribute to tumor development, progression and metastasis. This is particularly relevant given that survivin is considered a specific Tumor-Associated-Antigen (TAA) because the protein is overexpressed in most human cancers, but essentially absent in the respective normal tissues, although exceptions do exist [2, 89].

In a meta-analysis including 2703 patients with non-small cell lung cancer (NSCLC), survivin expression was identified as a factor indicative of poorer prognosis in advanced stages of NSCLC (stages III-IV) rather than early stages (I-II) [182]. In a meta-analysis involving 1365 gastric cancer patients, survivin expression was associated with worse overall survival. Specifically, cytoplasmic, but not nuclear, survivin expression was linked to a poorer prognosis for those patients. Hence, not only expression per se, but also the subcellular localization of survivin appears to be important in gastric cancer survival [183].

However, survivin expression is not necessarily always bad. In a study with 60 ovarian cancer patients at advanced stages (stages IIIC, IV FIGO classification) of disease, survivin and p53 expression were analyzed before and after neoadjuvant chemotherapy [184]. Nuclear survivin expression was detected in almost 60 % of patients before treatment, and after neoadjuvant chemotherapy, nuclear survivin expression was reduced. Furthermore, elevated nuclear survivin expression was identified as a favorable prognostic marker in patients treated with neoadjuvant chemotherapy. The median overall survival for p53 positive patients with higher expression of nuclear survivin was 34.6 months, compared to 22.2 months for those patients with lower nuclear survivin expression [184]. These observations implicate nuclear survivin expression as a favorable prognostic marker for chemotherapy in patients with advanced ovarian cancer [184]. This will become important subsequently due to the relevance of angiogenesis in ovarian cancer progression [185, 186], and the increase in vasculogenic mimicry detected in ovarian cancer patients [1, 105].

For the reasons mentioned, there has been great interest in developing approaches that seek to reduce survivin expression in order to limit cancer cell growth, as will be eluded to in the subsequent section. However, as the previous paragraph indicates, survivin expression, particularly in the nucleus, can also be beneficial to patients, thus complicating the expected outcome of such treatments.

### Survivin as a target in cancer therapy

Survivin is a member of the IAP family, of which several members are deregulated in human cancers, including solid tumors and hematological malignancies [90, 187–189]. Consistent with these observations, targeting other IAPs in combination with cytotoxic drugs has been suggested as a treatment for hematological malignancies [188]. However, strategies focusing on survivin are generally favored over the targeting of other IAPs because survivin expression is fairly specific, although not exclusive to tumor cells and because survivin displays characteristics of a nodal protein by participating in a great variety of pathways and processes that favor tumor cell development [90]. For precisely these reasons, survivin has been widely exploited as a pharmacological target in cancer (Table 1). Multiple strategies are currently being evaluated in clinical trials, including the use of survivin inhibitors and the development of survivin-based vaccines (Table 1).

**Table 1:** Clinical trials targeting survivin in cancer

Strategy	Pathology	Phase	Clinical trials identifier
Survivin inhibitors			
YM155 (survivin suppressor) together with Paclitaxel and carboplatin	Solid tumors and advanced non-small cell lung carcinoma	I/II	NCT01100931
Terameprocol (EM1421), inhibitor of survivin and cdc2 (cyclin-dependent kinase-1) in continuous intravenous infusion	Refractory Solid tumors	I	NCT00664586
EZN-3042, a locked nucleic acid antisense oligonucleotide	Acute Lymphoblastic Leukemia	I	NCT01186328
Terameprocol (EM-1421), inhibitor of survivin and cdc2 (cyclin-dependent kinase-1)	Leukemia	I	NCT00664677
EZN-3042, a survivin-targeted mRNA antagonist, alone or in combination with standard chemotherapy	Acute Lymphoblastic Leukemia	I	NCT01186328
LY2181308, an antisense oligonucleotid, targeted against survivin mRNA in combination with idarubicin and cytarabine	Acute Myeloid Leukemia	II	NCT00620321
Survivin-based Cellular Therapy			
Dendritic cell vaccine (mRNA from PSA, PAP, survivin and hTERT) plus docetaxel or docetaxel alone	Prostate Cancer (castration resistant and metastatic)	II	NCT01446731
Dendritic cells - transfected with hTERT-, survivin- and tumor cell derived mRNA + ex vivo T cell expansion and reinfusion	Melanoma	I/II	NCT00961844
Drug: Temozolomide			
Procure®, denditric cells loaded with Survivin-peptide and Telomerase mRNA	Ovarian Cancer	I	NCT01456065
Dendritic cell loaded with amplified ovarian cancer stem cell mRNA, hTERT/survivin mRNA	Ovarian Cancer	I/II	NCT01334047
Cell therapy based on dendritic cells transfected with Survivin, hTERT and p53 mRNA	Metastatic breast cancer	I	NCT00978913
	Malignant melanoma		
TAA-SPECIFIC CTLs targeting survivin, PRAME, NY-ESO-1, MAGEA4 and SSX	Solid Tumors (TACTASOM)	I	NCT02239861
Treatment with autologous dendritic cells transfected with Survivin, MelanA and MAGE-A3 mRNA or loaded with MAGE-A3, MelanA and Survivin	Melanoma	I/II	NCT00074230
Cell therapy with cytotoxic T lymphocytes exposed to tumor associated antigens: NY-ESO-1, MAGEA4, PRAME, Survivin and SSX.	Hodgkin or Non-Hodgkin Lymphoma	I	NCT01333046
TAA-CTLs may be generated from donors or recipients and will be tested for specificity against 4 tumor antigens commonly found in hematological malignancies (WT1, PRAME, SURVIVIN, and MAGE-A3).		I	NCT02203903
	Hematological		
	Malignancies		
Dendritic cell vaccine (MUC-1 and survivin) in combination with cytokine-induced killer cells	Soft Tissue Sarcoma	I/II	NCT01898663
Dendritic cell vaccine (MUC-1 and survivin) in combination with cytokine-induced killer cells	Renal Cell Carcinoma	I/II	NCT01924156
Autologous dendritic cell vaccine (peptides from survivin and telomerase)	Renal Cell Carcinoma	I/II	NCT00197860
Multiple antigen specific cellular therapy: autologous T cytotoxic cells induced by dendritic cells (loaded with p53, her2, survivin and a total of 17 antigens)	Hepatocellular Carcinoma	I/II	NCT02026362
Vaccine therapy (p53, survivin and telomerase) with autologous dendritic cells in combination with adjuvant cytokines	Advanced Melanoma	I/II	NCT00197912
Multiple tumor-associated antigen (TAA)-specific T cells (against WT1, PRAME and survivin) from donors	Acute Lymphoblastic Leukemia	I	NCT02475707
Multiple tumor-associated antigen (TAA)-specific T cells (against WT1, NY-ESO-1, PRAME and survivin) from donors	Acute Lymphoblastic Leukemia/Myelodisplasic Syndrome	I	NCT02494167
Multiple tumor-associated antigen (TAA)-specific cytotoxic T cells (against NY-ESO-1, IMAGEA4, PRAME, SSX and survivin) from donors	Multiple Myeloma	I	NCT02291848
Survivin-vaccines			
Survivin peptide vaccination in combination with sargramostin	Malignant Glioma	I	NCT01250470
Vaccination with DPX-Survivin in combination with low doses of cyclophosphamide (The peptide antigens targeting survivin)	Advanced Stage Ovarian, Fallopian or Peritoneal Cancer	I/II)	NCT01416038
hTERT/survivin/CMV multipeptide vaccine	Breast Cancer	Not provided	NCT01660529
Multipeptide vaccination including survivin-peptide	Multiple Myeloma	I/II	NCT00499577
SurVaxM Vaccine (survivin-peptide vaccine) in combination with temozolamide	Glioblastoma	II	NCT02455557
Peptide vaccine (IDO/survivine peptide) as enhancer of temozolomide chemotherapy	Metastatic Melanoma	II	NCT01543464
Vaccine therapy (MART1 analog, gp100 and survivin) and GM-CSF with or without Aldesleukin	Melanoma	I	NCT00470015
Vaccine therapy (survivin) in patients receiving lenalidomide	Multiple Myeloma	I	NCT02334865
hTERT tumor vaccine (peptides from telomerase, survivin and cytopeptide) in combination with autologous T cell infusion	Multiple Myeloma	I/II	NCT00834665
Immunotherapeutic vaccine DPX-Survivac (targeting survivin) in combination with cyclophosphamide	Diffuse Large B-Cell Lymphoma	II	NCT02323230
Survivin and telomerase peptide vaccination in combination with Daclizumab and Prevnar	Advanced Breast cancer	I	NCT00573495
Survivin peptide vaccination	Advanced Melanoma, pancreatic, colon and cervical cancer	I/II	NCT00108875

Unfortunately, studies focusing on pharmacological inhibitors of survivin have shown rather disappointing results. In a phase I clinical trial in patients with acute lymphoblastic leukemia, EZN3042, a locked antisense construct against survivin, was toxic and patients showed poor tolerance to treatment [190]. Subsequently, the application of EZN3042 was suspended for the indicated reasons [190]. In a study in patients with advanced NSCLC solid tumors, YM155, an inhibitor of Sp1-mediated survivin expression, displayed an acceptable safety profile; however, this compound failed to improve responses to chemotherapy treatment [191]. In patients with leukemia, Terameprocol, an inhibitor of survivin and cyclin-dependent kinase-1 was found to be safe in phase I study. In addition, a therapeutic effect (partial response and disease stabilization by terameprocol) was observed in patients treated with this compound [192]. Currently, an additional trial is underway evaluating terameprocol in patients with refractory solid tumors (Clinical trial identifier NCT00664586). Additional studies are required to corroborate the utility of these approaches in cancer treatment.

Multiple survivin-based studies involving cell therapy are currently underway. In this context, the use of dendritic cells loaded with survivin-peptide (Clinical trial identifier NCT01456065) or survivin mRNA (Clinical trial identifier NCT01334047, NCT00978913) in association with telomerase and p53 mRNAs are being evaluated in clinical trials in patients with ovarian cancer, metastatic breast cancer and malignant melanoma. Furthermore, the efficacy of cytotoxic T lymphocytes exposed to a mixture of TAAs, including survivin, is currently being tested in the treatment of hematological malignancies (clinical trial identifier NCT01333046, NCT02203903, NCT02475707). Thus, cellular therapy represents an intense area of contemporary research, although clear benefits of such treatments remain to be established.

In addition to the approaches mentioned, considerable effort is being placed on the development of survivin-based vaccines (survivin mRNA and peptide) for the treatment of several different types of cancer, including breast cancer, kidney cancer, advanced melanomas and ovarian cancer (Table 1). Promising results were obtained in renal carcinoma patients where survivin-vaccination lead to disease stabilization [193] and a response in 35 % of the patients without adverse toxicity effects [194]. The most successful results have been obtained in ovarian cancer, where DPX-Survivac, a vaccine based on the use of survivin peptides in conjunction with a DepoVax™ adjuvant, administrated in a treatment together with cyclophosphamide, yielded favorable results in a phase I clinical trial. This treatment was found to be safe, well-tolerated by patients and yielded strong immune responses against tumors [195]. Recently, DPX-Survivac was designated by the FDA as an orphan drug for maintenance therapy in ovarian cancer patients with no measurable disease after standard treatments (surgery/chemotherapy). These promising results obtained with DPX-Survivac open up a wide array of possibilities and further studies are required to determine the efficacy of this vaccine in phase II trials.

In a phase II trial in patients with metastatic melanoma, a treatment involving vaccination with autologous dendritic cells previously pulsed with survivin, hTERT and p53-derived peptides together with cyclophosphamide and celecoxib (COX-2 inhibitor) was evaluated (clinical trial identifier NCT00197912) [196]. This treatment was shown to be safe and tolerable and an increase in the immune response was detected. Also, for almost 60 % of patients the disease was stabilized for four or more months (clinical trial identifier NCT00197912) [196]. In summary, many clinical trials are currently underway to determine whether targeting survivin represents an effective approach to limit tumor development. Although some trials have unfortunately met with limited success, others, such as those targeting ovarian cancer, have yielded highly promising results. Here, it should be noted that ovarian cancer is precisely a case where angiogenesis represents a highly prevalent “hallmark” trait, underscoring thereby the importance of survivin in this context, as has been discussed throughout this review.

## Conclusions

Survivin plays an important role in processes that favor tumor growth and angiogenesis. HIF1α stabilization under low oxygen conditions and/or via ROS production promotes survivin and VEGF expression and favors angiogenesis. In addition to the well-established role of survivin in endothelial cells, survivin in tumor cells enhances β-Catenin Tcf/Lef-dependent VEGF transcription, synthesis and release, thereby promoting angiogenesis of endothelial cells. More recently, cancer cells have also been shown to form vascular-like structures in the absence of endothelial cells in a process known as vasculogenic mimicry. In a poorly vascularized tumor microenvironment, we posit that hypoxia-enhanced survivin levels may increase VEGF production and EMT, thus promoting the process of vasculogenic mimicry. While highly intriguing, this possibility remains to date largely speculative; however, this should represent a fruitful area for research in the future, both in the perspective of developing a better understanding of the underlying mechanisms, as well as how such insight might be harnessed to treat tumors more effectively.
